# In Vivo, High-Throughput Selection of Thermostable Cyclohexanone Monooxygenase (CHMO)

**DOI:** 10.3390/catal10080935

**Published:** 2020-08-13

**Authors:** Sarah Maxel, Linyue Zhang, Edward King, Ana Paula Acosta, Ray Luo, Han Li

**Affiliations:** 1 Department of Chemical and Biomolecular Engineering, University of California, Irvine, CA 92697, USA; 2 Department of Molecular Biology and Biochemistry, University of California, Irvine, CA 92697, USA; 3 Department of Materials Science and Engineering, University of California, Irvine, CA 92697, USA

**Keywords:** cyclohexanone monooxygenase, *Acinetobacter* sp. NCIMB 9871, Baeyer–Villiger monooxygenase, directed evolution, thermostability, NADPH specificity, redox balance, high-throughput selection

## Abstract

Cyclohexanone monooxygenase (CHMO) from *Acinetobacter* sp. NCIMB 9871 is characterized as having wide substrate versatility for the biooxidation of (cyclic) ketones into esters and lactones with high stereospecificity. Despite industrial potential, CHMO usage is restricted by poor thermostability. Limited high-throughput screening tools and challenges in rationally engineering thermostability have impeded CHMO engineering efforts. We demonstrate the application of an aerobic, high-throughput growth selection platform in *Escherichia coli* (strain MX203) for the discovery of thermostability enhancing mutations for CHMO. The selection employs growth for the easy readout of CHMO activity in vivo, by requiring nicotinamide adenine dinucleotide phosphate (NADPH)-consuming enzymes to restore cellular redox balance. In the presence of the native substrate cyclohexanone, variant CHMO GV (A245G-A288V) was discovered from a random mutagenesis library screened at 42 °C. This variant retained native activity, exhibited ~4.4-fold improvement in residual activity after 30 °C incubation, and demonstrated ~5-fold higher cyclohexanone conversion at 37 °C compared to the wild type. Molecular modeling indicates that CHMO GV experiences more favorable residue packing and supports additional backbone hydrogen bonding. Further rational design resulted in CHMO A245G-A288V-T415C with improved thermostability at 45 °C. Our platform for oxygenase evolution enabled the rapid engineering of protein stability critical for industrial scalability.

## 1. Introduction

Baeyer–Villiger monooxygenases (BVMO) are valuable biocatalysts known for catalyzing the oxidation of (cyclic) ketones into esters and lactones through the utilization of reduced nicotinamide adenine dinucleotide (phosphate) (NAD(P)H). These enzymes have been studied for their industrial potential in the production of nylon monomers [1], (*Z*)-11-(heptanoyloxy)undec-9-enoic acid [2], and prostaglandins [3]. An NADPH-dependent BVMO, *Acinetobacter* sp. NCIMB 9871 cyclohexanone monooxygenase (*Ac* CHMO), has been extensively characterized in particular due to its versatile substrate scope and superior activity. However, one of the fundamental limitations to large-scale use of this enzyme is its well documented temperature instability [4,5].

To address the poor thermostability, two main strategies have been approached. First, there have been extensive efforts to expand the substrate scope of BVMOs which have good stability but relatively narrow substrate ranges [3,6–8]. Second, protein engineering efforts have been applied to make the broad substrate-range *Ac* CHMO less labile by introducing computationally predicted disulfide bonds [9,10] and creating chimeric enzymes between *Ac* CHMO and its more thermostable homologs [11].

Further engineering with these methods is constrained by the available sites for potential disulfide bond introduction and the limited substrate scope of the homologs [11]. While these rational methods discovered stability enhancing mutations, thermostability is a complex and global trait [12,13], and unexplored regions that affect thermostability are difficult to predict for engineering. A number of studies have used random methods effectively to discover critical unfolding regions and improve the thermostability [14–16]. To support random engineering, a high-throughput selection method is needed [10].

Recently, we described the construction and application of an aerobic growth-based, high-throughput selection platform for engineering NADPH-dependent oxygenases [17]. This selection platform is based on the principle of cofactor redox balance [17–22]: NADPH generation is increased in the engineered *E. coli* strain MX203 by re-routing glucose metabolism through the pentose phosphate pathway; in addition, NADPH recycling is blocked by disrupting NADPH:quinone oxidoreductase and transhydrogenase. As a result, the cells cannot grow unless a NADPH-consuming enzyme is introduced to restore the NADPH/NADP^+^ balance. This selection platform employs growth as an easily measurable readout and features a >10^6^ sample throughput per round. However, it has not been used at an elevated temperature to engineer enzyme thermostability.

**Figure 1.**
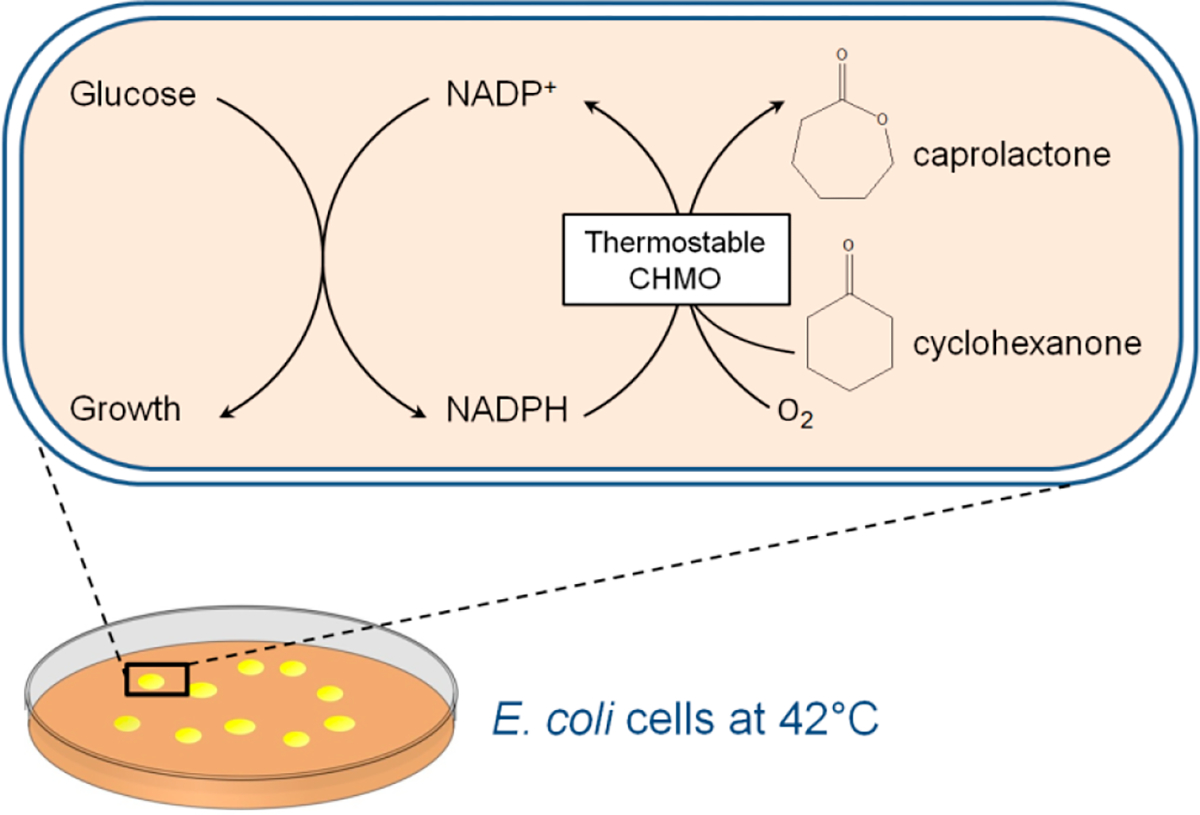
A redox balance-based selection platform at a high temperature. Growth restoration of selection stain MX203 is enabled by NADPH-consuming *Acinetobacter* sp. cyclohexanone monooxygenase (*Ac* CHMO), which converts cyclohexanone to caprolactone. At an elevated growing temperature at 42 °C, only thermostable CHMO variants will enable growth, which is easily identified by monitoring colony formation on agar plates.

Here, we sought to leverage the high throughput of the growth-based selection and mine beneficial mutations from a larger protein sequence space generated by error-prone PCR. Through a single round of selection at 42 °C (Figure 1), we discovered the variant CHMO GV (A245G-A288V) which retained ~83% activity after incubation at 30 °C, while the wild type retained only ~19%. Further rational design exploring the synergy between a previously reported stability-enhancing mutation [9] and our newly identified set in this study resulted in CHMO GV-T415C (A245G-A288V-T415C), which has further improved thermostability up to an even higher temperature of 45 °C. Compared to the previously reported most thermostable CHMO variant, the new variants reported here performed better in cyclohexanone conversion processes, reaching ~50% conversion which is 5-fold higher than what is achieved using wildtype CHMO. To understand the structural basis underlying CHMO GV’s enhanced thermostability, we performed molecular modeling which indicates that CHMO GV experiences more favorable residue packing and backbone torsions, which is difficult to design for without a high-throughput selection method.

## 2. Results

### 2.1. Identification of CHMO Variants with Increased Thermostability

**Figure 2.**
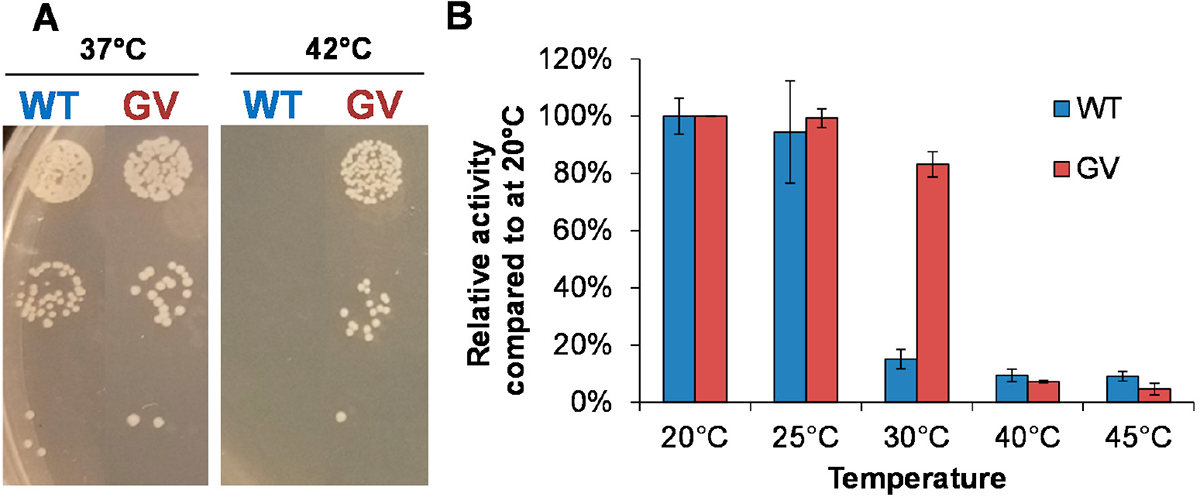
Analysis of CHMO GV with improved thermostability. (**A**) At 42 °C, CHMO GV, but not wild type (WT), restores growth of the selection strain MX203; (**B**) CHMO GV retains higher percentage of activity than WT after 10 min of 30 °C temperature exposure. Both variants experience substantial activity loss after incubation at 40 °C and 45 °C. Values are an average of two or three replicates, and the error bars represent one standard deviation.

CHMO wild type (WT) can restore growth of the NADPH-dependent selection strain (MX203) at 37 °C in minimal medium on glucose, but failed to restore the growth at 42 °C (Figure 2A), suggesting that the activity level of CHMO WT is considerably reduced at elevated temperatures, which is consistent with its well documented poor thermostability [4,5].

To improve CHMO thermostability, we sought to introduce random mutations using error-prone PCR. A mutation rate of one to two mutations per variant was targeted during the construction of the CHMO library (pLS303. Table S1) [23]. A library size of roughly 2.4 × 10^7^ variants was subjected to selection in strain MX203 on agar plates with 2 g/L D-glucose in M9 minimal medium and 2 g/L cyclohexanone at 42 °C. The selection resulted in a single colony with superior growth, while ~18 additional colonies were observed with significantly slower growth. Due to the drastic growth difference between colonies, only the single fastest growing colony was extensively characterized. Sequencing of this variant revealed two mutations, A245G and A288V, previously unidentified as relevant to protein stability. The double mutant *Ac* CHMO A245G-A288V (CHMO GV) demonstrated reliable growth restoration of strain MX203 at 42 °C (Figure 2A). CHMO GV was purified and characterized in vitro alongside CHMO WT (Figure 2B). CHMO GV exhibited higher thermostability than WT: After a 10-min incubation at 30 °C, the WT retained only ~19% activity while GV retained ~83%. While having improved thermostability, CHMO GV also exhibited comparable catalytic efficiency compared to CHMO WT (Table 1).

### 2.2. The Effects of Thermostability Enhancing Mutations on Protein Dynamics

**Figure 3.**
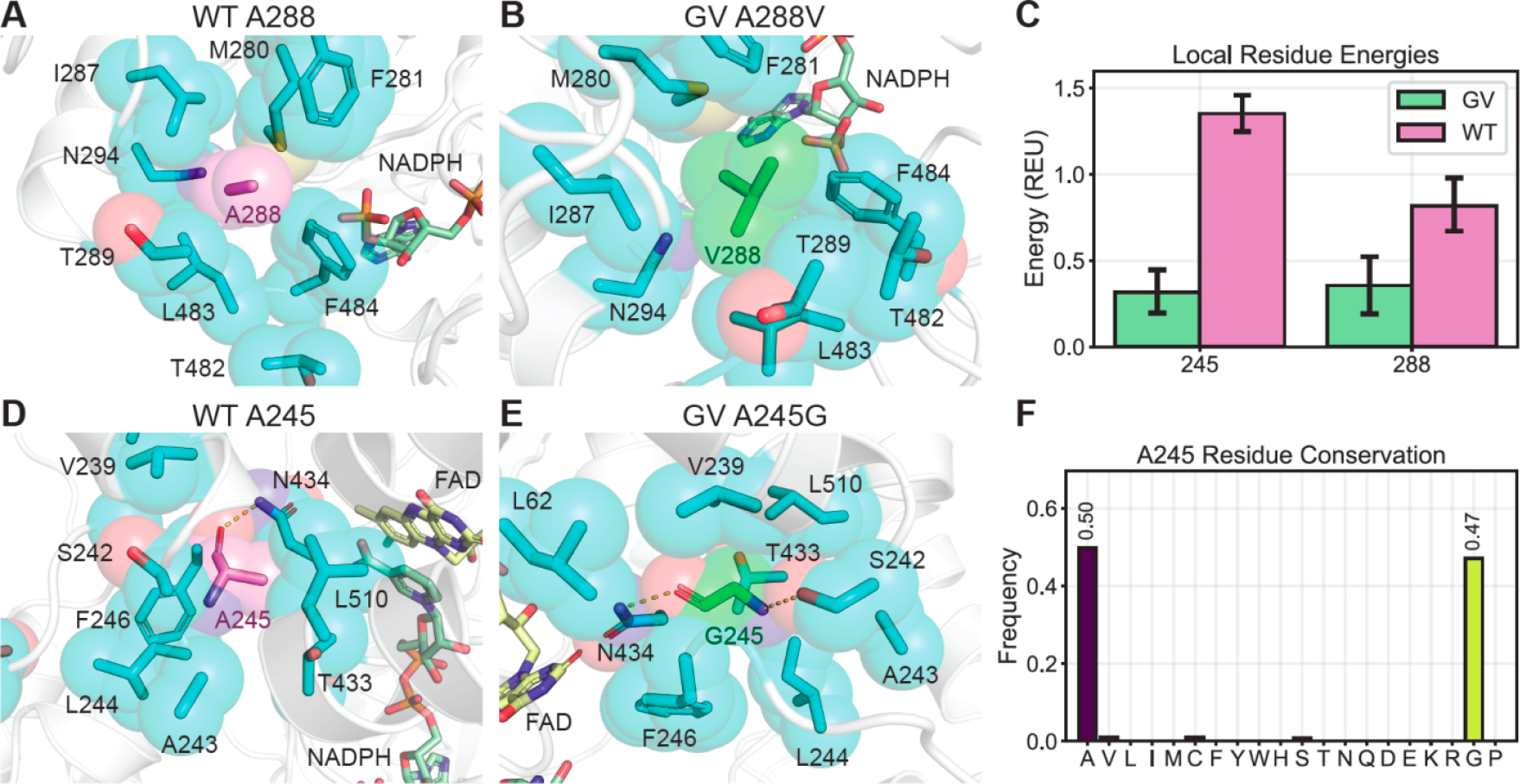
Molecular dynamics (MD) conformational analysis indicates that more optimal residue packing and backbone torsion underlies the increased thermostability of CHMO GV. (**A**,**B**) At position 288, valine has greater volume and surface area to maximize hydrophobic contact with surrounding residues; (**C**) Rosetta energy evaluation of snapshots from the MD trajectories indicates that interacting residues surrounding the mutated positions experience more favorable contacts in CHMO GV compared to WT; (**D**,**E**) At position 245, glycine is better enveloped by nearby residues, and has greater backbone torsion freedom to form an additional backbone hydrogen bond. (**F**) Measurement of residue frequencies from sequence alignment of *Ac* CHMO homologs shows that Ala and Gly are highly conserved at position 245. Values are an average of 200 frames and error bars represent 95% confidence intervals with 1000 bootstrap iterations.

We examined the impact of the GV mutations on CHMO conformational dynamics to understand how they contribute to enhanced thermostability. Since there are no crystal structures of *Ac* CHMO with cofactors bound available, we constructed a homology model with NADPH and flavin adenine dinucleotide (FAD) bound in the *Ac* CHMO structure using Rosetta Comparative Modeling, based on *Rhodococcus* sp. CHMO [24], which shares 57.8% sequence identity. We ran 400 ns molecular dynamics (MD) trajectories of CHMO GV and CHMO WT, and featurized the trajectories on minimum heavy atom distance from residues within 5 Å of position 245 or 288, followed by dimensionality reduction with principle component analysis (PCA). The conformations were discretized with K-means clustering in PCA space to identify the most populated metastable states for analysis (Figure S1). We visualized the frames with minimum distance to the cluster center as the representative conformations involving positions 245 and 288 (Figure 3A,B,D,E). We further extracted 200 frames from the most populated cluster of each sample, and evaluated the local structure stability as a function of the local Rosetta energy, which is the sum of residue energies measuring the favorability of steric, geometric, and electrostatic interactions for all amino acids within 5 Å of positions 245 and 288 (Figure 3C).

In CHMO WT, A288 leaves excess space for water molecules to enter and causes undesired side-chain flexibility (Figure 3A). In contrast, A288V in CHMO GV completely fills the small void created by the residues nearby and provides a greater nonpolar surface area to encourage closer packing (Figure 3B). As a result, CHMO GV exhibits greater stability at position 288, scoring 0.35 ± 0.2 Rosetta Energy Units (REU), compared to 0.81 ± 0.2 REU for CHMO WT (Figure 3C). At position 245, the reduced bulk from A245G allows the surrounding residues to pack more tightly in CHMO GV with fewer gaps for solvent to enter (Figure 3D,E). More importantly, the increased torsion flexibility from glycine permits A245G in CHMO GV to contort and form backbone hydrogen bonds with N434 and S242. The more restricted backbone torsions due to the alanine methyl sidechain steric interactions prevent A245 in CHMO WT from satisfying both backbone hydrogen bonds simultaneously (Figure 3D,E). These interactions are reflected by the local energy scores (Figure 3C), with CHMO GV scoring more favorably than CHMO WT at position 245 (0.32 ± 0.1 REU versus 1.35 ± 0.1 REU).

Interestingly, bioinformatic analysis of natural residue diversity at A245 in *Ac* CHMO homologs indicates that alanine and glycine are sampled in 97% of homologous sequences, with glycine in 47% of sequences and alanine in 50% (Figure 3F). Comprehensive residue frequency analysis is described in the Supplementary Information (Figures S2 and S3) in addition to the complete homolog list (Table S2). Our selection method which had access to the full set of amino acids converged to the same highly conserved residue collection as Nature, indicating that there is a strong selective pressure to maintain small amino acids at position 245, and that our selection explores sequence space in a manner analogous to natural evolution.

### 2.3. Further Improving Thermostablity by Rational Design

**Figure 4.**
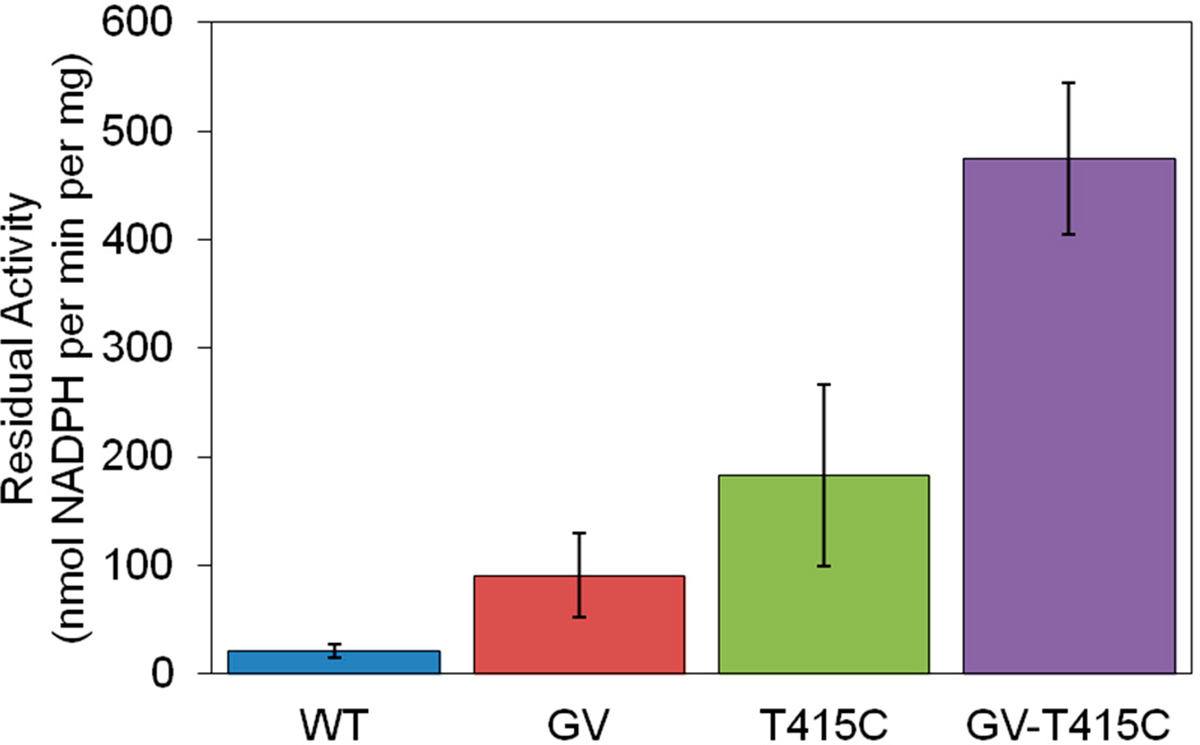
Residual specific activity of CHMO variants after ten-minute incubation at 45 °C. Variants GV and T415C displayed 4.4 and 8.8-fold improved residual activity over WT. Variant combining the stabilizing mutations GV and T415C dramatically increased residual activity 23-fold suggesting a synergistic effect on protein stability.

CHMO GV still lost substantial activity after incubation at 40 °C and 45 °C (Figure 2B). We sought to explore potential synergy between the mutations in variants GV and T415C, a single-point mutant described by Schmidt et al. with significant improvements in long-term stability [9]. Introduction of T415C, a free cysteine, in addition to selected mutations, yields a triple mutant with greatly improved activity and stability at 45 °C relative to variant GV or T415C alone. The triple mutant CHMO GV-T415C has 23-fold higher activity compared to WT after 10-min incubation at 45 °C (Figure 4).

The additive effect observed for the three distal mutations is likely a consequence of their spatial isolation, where changes independently address local instabilities that could result in protein unfolding [25]. This suggests that further beneficial mutations can be obtained by iterative rounds of random mutagenesis.

### 2.4. Cyclohexanone to Caprolactone Conversion Using Thermostable CHMO

**Figure 5.**
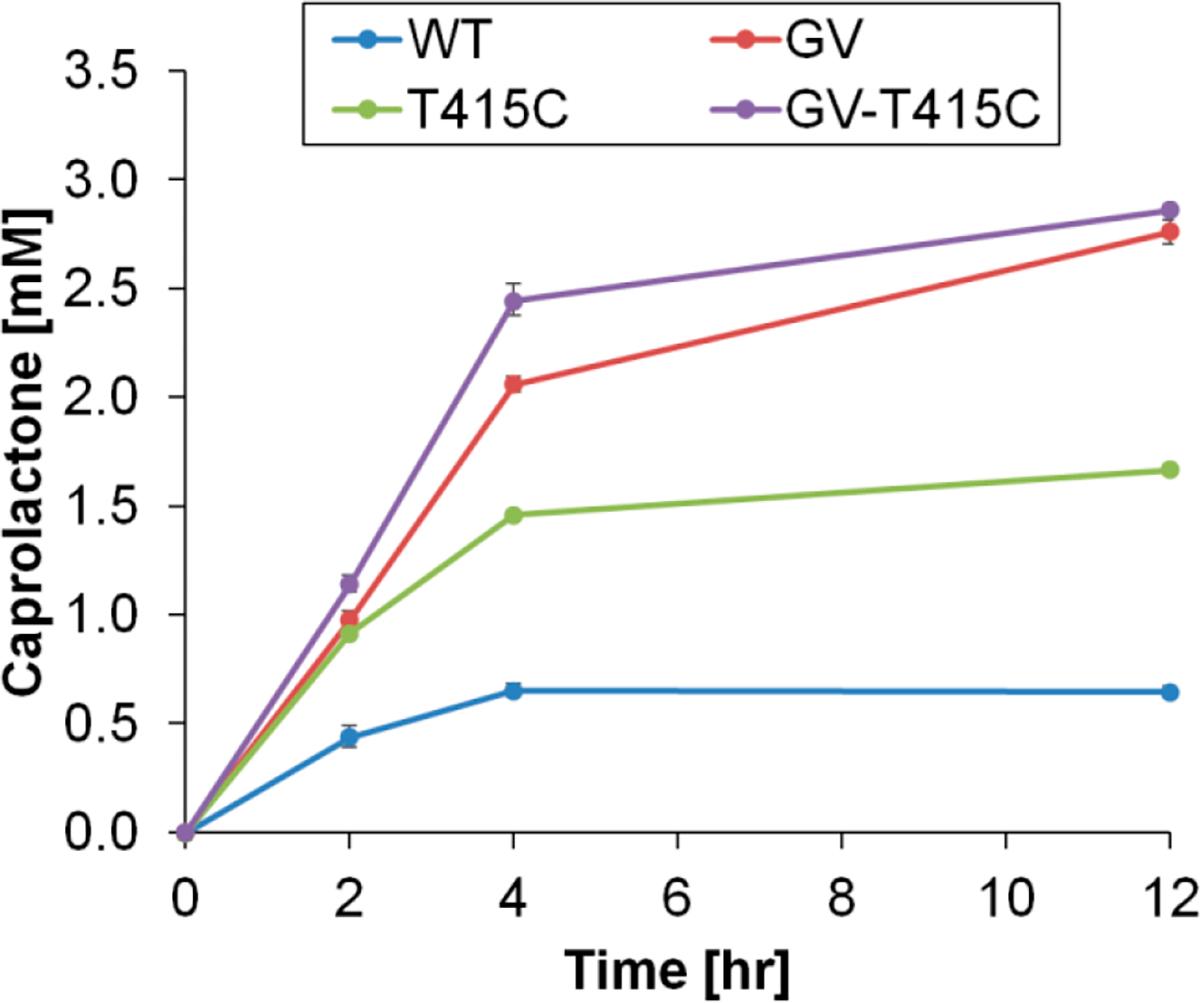
CHMO panel conversion of cyclohexanone to caprolactone in a standard reaction mixture (5 mM NADPH, 5 mM cyclohexanone, 50 mM sodium phosphate pH 7.7, 6 μg/mL purified protein). Reaction was monitored over 12 h at 37 °C. The variant obtained from this study, CHMO GV (red), supported improved conversion over WT CHMO (blue) and CHMO T415C (green). While WT CHMO activity diminished greatly over 4 h incubation and converted only ~10% of substrate available, both CHMO GV-T415C (purple) and CHMO GV retained consistent conversion during 4 h period and achieved ~50% conversion after 12 h incubation. Values are an average of three replicates, and the error bars represent one standard deviation.

To test thermostable CHMO variants for their potential application as industrial catalysts, we performed conversion assays with 5 mM cyclohexanone as the substrate, 5 mM NADPH as the co-substrate, and at 37 °C, a commonly used temperature in biotransformation (Figure 5). CHMO WT only produced ~0.5 mM product caprolactone (~10% conversion) because the activity diminished after 4 h, likely due to the enzyme’s poor thermostability. The thermostable variant identified from growth selection, CHMO GV, produced ~2.5 mM caprolactone in 12 h, reaching ~50% conversion. CHMO GV also performed better than the previously reported thermostable CHMO variant, CHMO T415C [9].

Interestingly, despite its higher thermostability (Figure 4), the triple mutant CHMO GV-T415C conferred a nearly identical final conversion to that of CHMO GV (Figure 5). These results suggest that other limiting factors may exist apart from enzyme thermostability. One hypothesis is that cofactor stability may be limiting, as we observed appreciable NADPH degradation at 37 °C after 4 h. In agreement with this hypothesis, we observed a faster conversion rate with CHMO GV-T415C compared to CHMO GV during the initial 4 h, but this trend could not be sustained. Future work is needed to test this hypothesis and further optimize the process. For example, supplementation of fresh NADPH or a cofactor recycling enzyme such as *Bacillus subtilis* glucose dehydrogenase [26] may be incorporated to maintain cofactor availability.

## 3. Discussions

The growth-based platform MX203 enabled the efficient isolation of the stabilizing mutation pair A245G-A288V. This in vivo approach provides a simple means to select for enzymes with improved thermostability and kinetic parameters relevant to physiological conditions, which is useful for potential in vivo applications and for avoiding variants with impractically high K_M_ values. As shown in Table 1, CHMO GV has similar K_M_ for NADPH compared to that of the wild type. In contrast, in vitro selections often select for variants with non-physiologically relevant substrate affinities because the selections themselves are performed at elevated cofactor and substrate concentrations to ensure high sensitivity for detecting activity. CHMO GV highlights the capability of growth-based selections in rapidly identifying productive mutations that improve enzyme stability without deterring downstream industrial potential.

Despite the high throughput of the method, only one variant capable of restoring robust growth was obtained from the Error-Prone PCR Library generated in this study after the 42 °C incubation. Although the expansive sequence space available in the random library was not completely covered during selection, increasing the scale of the library transformation can supplement coverage. In addition to improved stability, the selection platform MX203 requires the variants to provide sufficient NADPH recycling to restore growth; within the library tested it is possible that these selection criteria were not met by the remaining variant pool to enable growth at 42 °C. Although further selections at intermediate incubation temperatures could reveal moderately stabilizing mutations, the generation and selection of a semi-rational library might provide more insight into general approaches for improving thermostability.

Optimal hydrophobic packing through complementary steric interactions and burial of hydrophobic residues is associated with higher protein thermostability [27], but it has been difficult to apply this design principle in rational engineering because sites to target and substitutions to introduce are challenging to predict. Computational methods can identify voids in proteins and facilitate design to reduce packing defects [28], but often ignore the roles of the residues in the folding pathway [29], neglect the impact of predicted mutations on catalytic function [30], overlook dynamical changes in protein conformation that alter the local environments surrounding each residue [31], and coarsely survey discrete rotamer states with minimal backbone flexibility [32], precluding the accuracy necessary to achieve ideal packing. Semi-rational methods still require numerous candidates to be tested experimentally, which is resource intensive and time consuming. Our growth selection platform overcomes this sampling problem because its high throughput supports random engineering to broadly explore sequence space, and directly yields active variants.

The current platform constructed in mesophilic *E. coli* is limited by growth inhibition at temperatures exceeding 42 °C. Construction of a similar selection strain with co-expression of auxiliary heat shock proteins such as Oshsp16.9 [33], in a heat resistant *E. coli* with a maximum growth rate at 48.5 °C [34], or in a thermophilic bacteria such as *Geobacillus thermoglucosidasius* [35] may allow facile identification of increasingly thermostable variants.

## 4. Materials and Methods

### Media and Growth Conditions.

Cloning was carried out with *E. coli* XL-1 blue and protein expression was performed with MX203 as indicated below. All *E. coli* were cultured in 2xYT containing 16 g/L Tryptone, 10 g/L Yeast Extract and 5 g/L NaCl unless otherwise noted. Super Optimal Broth (SOB) media consisted of 20 g/L Tryptone, 5 g/L Yeast Extract, 0.5 g/L NaCl, 4.94 g/L MgSO_4_· 7H_2_O, and 0.2 g/L KCl. Super Optimal Broth with Catabolite Repression (SOC) media shared the same composition of SOB media with the inclusion of 20 mM D-glucose. M9 Wash Buffer consisted of 1 mM MgSO_4_, 0.1 mM CaCl_2_, trace metal mix A5 with Co (H_3_BO_3_ 2860 μg/L, MnCl_2_ · 4H_2_O 1810 μg/L, ZnSO_4_ 7H_2_O 222 μg/L, Na_2_MoO4, 2H_2_O 390 μg/L, CuSO_4_, 5H_2_O 79 μg/L, Co(NO_3_)_2_·6H_2_O 49 μg/L, and BD Difco M9 salts (Na_2_HPO_4_ 6.78 g/L, KH_2_PO_4_ 3g/L, NaCl 0.5 g/L, NH_4_Cl 1 g/L). M9 Selection Media shared the same composition as M9 Wash Buffer with the inclusion of 2 g/L D-glucose, 0.01 g/L thiamine, 0.04 g/L FeSO_4_ · 7H_2_O. For solid media M9 Selection Plates, 15 g/L agar was added in addition to M9 Selection Media composition. In overnight cultures, the substrate cyclohexanone was added at a concentration of 1 g/L and in growth restoration experiments 2 g/L was added from a concentrated stock when appropriate. Concentrations for antibiotic selection were 100 mg/L for ampicillin, 50 mg/L for kanamycin, 50 mg/L for spectinomycin, and 10 mg/L for tetracycline. All strains were cultured at 37 °C with 250 r.p.m agitation unless otherwise noted. Induction in overnights was initiated with final concentrations of 0.01% arabinose for strains with arabinose-inducible promoter.

### Plasmid Construction and Strain Construction.

All PCR fragments were generated using PrimeSTAR Max DNA Polymerase (TaKaRa) unless otherwise noted. Splicing-by-overlap extension (SOE) PCR and degenerate codon PCR was conducted using KOD Xtreme Hot Start DNA Polymerase (Novagen). The *Acinetobacter* sp. NCIMB 9871 *chnB* gene was amplified from an *E. coli* codon optimized gBlock (IDT). After PCR and gel extraction, the *Ac chnB* gene fragment was inserted into the pRSF vector backbone which contained a 6×His tag at the C-terminus, using the Gibson isothermal DNA assembly method, resulting in plasmid pLS301. Plasmid pLS302 was generated using pLS301 as a template and applying site directed mutagenesis to introduce mutation T415C. Plasmid pLS305 was generated by the addition of mutation T415C using pLS304 as a template. Construction of strain MX203 was described previously [17].

### Generation of Error Prone PCR Library.

The *Ac* CHMO Error Prone PCR Library was cloned using the GeneMorph II Random Mutagenesis Kit (Agilent) to generate 1 to 2 mutations on gene *Ac chnB*. Using 180 ng of template DNA (provided by plasmid pLS301, Figure S4), the gene insert was amplified via error prone PCR with 25 reaction cycles according to manufacturer’s instructions. A panel of starting template material (150 ng to 250 ng) was initially evaluated to tune final range of mutations observed. After PCR, the gene fragment was digested overnight with restriction enzymes BamHI-HF and SalI (NEB) in parallel with the backbone fragment (NADH oxidase plasmid pLS101 provided pRSF backbone template for library construction). Gel fragments containing the target library insert and pRSF backbone (Figure S5) were recovered and concentrated using Zymoclean^™^ Gel DNA Recovery Kit (Zymo). Purified library inserts and backbone fragments were ligated overnight and then transformed into ElectroMAX DH10β competent cells (Invitrogen) by electroporation. Transformed cells were rescued in 600 μL SOC medium by shaking for 1 h at 37 °C. After rescue, cells were added to 20 mL 2xYT medium in a 250 mL baffled shake flask and 2 μL, 20 μL, and 200 μL was plated on 2xYT agar plates with appropriate antibiotics. Colonies formed on these plates were counted to estimate library size, 2.43 × 10^7^. The liquid culture was incubated at 37 °C for 10 h before plasmid extraction to generate the plasmid library, pLS303. Ten colonies from the library estimation plates were cultured in liquid medium for plasmid extraction and sequencing to confirm low mutation rate.

### Growth Rescue Conditions.

The growth rescue condition used is as follows: Briefly, the strains tested were first cultured in 2xYT under aerobic conditions at 30 °C overnight with appropriate antibiotics and inducers. Next, overnight cultures were washed 3 times and re-suspended in M9 Wash Buffer. For solid growth, targeted serial dilutions of 10^5^ cells/mL, 10^4^ cells/mL, and 10^3^ cells/mL were prepared in M9 Wash and 5 μL aliquots were dispensed in series on an agar plate of M9 Selection Media, with appropriate antibiotics and inducers. Plates were grown at designated temperatures and photos were taken to document growth progress. Plates were sealed with parafilm to minimize evaporation of substrate and media. This procedure was applied after selection to verify that variant expression conferred growth restoration.

### Transformation and Selection of *Ac* CHMO epPCR Library.

To generate electro-competent *E. coli* MX203 cells, a selection strain cultured in 200 mL SOB medium with appropriate antibiotics at 30 °C with shaking at 250 r.p.m. until OD_600nm_ reached 0.4–0.6. The culture was chilled on ice for 15 min and the cells were pelleted at 4 °C, 4000× *g*. The cells were washed at 4 °C three times with 40 mL 10% glycerol solution (ice cold). After, cells were finally resuspended with 500 μL 10% glycerol solution (ice cold), and aliquoted for transformation.

### The transformation was performed as follows:

After electro-competent cells were prepared, 20 μL of library DNA (pLS303) was added to 200 μL competent cells. Cell-DNA mixture was added to four ice chilled 1 mm gap electroporation cuvettes (55 μL per electroporation cuvette). Cells were electroporated at 2 kV, 129 Ω, 50 μF, resistance 2.5 kV; 200 μL of SOC medium was immediately added and transferred to a microcentrifuge tube at room temperature. This step was repeated twice more. Cells were rescued at 37 °C with shaking for 1 h. After that, cultures were combined in 20 mL 2xYT with appropriate antibiotics in a 250 mL baffled shake flask while controls were added to 5 mL 2xYT in a 50 mL conical tube (cap loose to allow increased aeration). Serial dilution of the library culture was performed and then plated on 2xYT agar plates with appropriate antibiotics. After incubation at 37 °C overnight, colonies formed were counted to estimate transformation efficiency, ~1 × 10^6^. Remaining cultures were grown at 30 °C for ~5 h or until OD_600nm_ = 0.6 was reached. Subsequently, controls and the library were induced by addition of arabinose. Cultures were grown for an additional 4 h (until OD_600nm_ = ~2.2).

To prepare cells for the selection condition, 1 mL of each culture was pelleted in 2 mL microcentrifuge tubes and washed three times with M9 Wash Buffer. After wash, cells were finally re-suspended in 1 mL M9 Wash Buffer. Cells were diluted with M9 Wash Buffer to a final cell concentration of ~10^7^ cells/mL. Twenty 100 μL aliquots of this cell suspension were plated on separate M9 Selection Plates supplemented with 2 g/L cyclohexanone and incubated at 42 °C for 48 h. Expression of CHMO WT from plasmid pLS301 was observed in parallel at 37 °C to serve as a positive control and at 42 °C to serve as a negative control. The colony growth was monitored periodically. A single colony was observed with rapid growth comparable to the positive control, while ~18 additional colonies were observed with significantly slower growth. Selected colonies were picked and re-streaked onto the fresh selection media and again incubated at 42 °C to obtain single colonies. Single colonies were cultured in liquid media to extract plasmids using the QIAprep Spin Miniprep kit (Qiagen) to yield pLS304. Due to the drastic growth difference between colonies, only the single fast-growing colony was extensively characterized.

### Determination of pLS302 Library Size Capability.

After electroporation and rescue, all transformants containing library plasmid were added to a flask containing 20 mL 2xYT. From this culture, 2 μL was plated and incubated overnight. The number of isolated MX203 colonies formed on this plate was counted as 50 colony forming units (CFU). The library size contained in the entire culture was calculated using Equation (1).


(1) $$Library\;Capability\;=\frac{50\;\text{CFU}}{2\;\mu \text{L}}\times \frac{1000\;\mu \text{L}}{\text{mL}}\times 20\;\text{mL}\approx 1\times 10^{6}\;individual\;trans\;formants$$

### Expression and Purification of CHMO Wild-type and Variants.

Enzymes purified for residual specific activity assay (Figure 2B) were expressed in *E. coli* strain BW25113 [36] incubated at 30 °C after induction. Larger-scale purification for enzymes applied in follow-up specific activity assay (Figure 4) and conversion assay used *E. coli* strain MX203 [17] for expression and cultures were incubated at 25 °C. Expression was carried out by inoculation of 50 mL 2xYT media supplemented with 100 μg/mL spectinomycin and an overnight culture. Cells were grown at 37 °C in baffled shaking flasks and were induced at an OD_600nm_ of 0.6–0.8 with 0.2% arabinose. The 50 mL main culture was then incubated at 25 °C or 30 °C and 250 r.p.m for 24 h. The expression was stopped by harvesting the cells (centrifugation at 4 °C, 4000 g for 15 min). Cell pellet was resuspended in HisPur^™^ Ni-NTA equilibration buffer (Thermo Fisher Scientific, Waltham, MA, USA) and mixed with 1 mL of 0.1 mm glass beads (Biospec, Berlin, Germany) and homogenized using a benchtop homogenizer (FastPrep-24, MP Biomedicals, Santa Ana, CA, USA). Cell debris was separated from the crude extract by centrifugation at 4 °C, 20,000 g for 15 min. Protein purification was performed using the HisPur^™^ Ni-NTA Protein Miniprep (Thermo Fisher Scientific) according to the manufacturer’s instructions. The histidine-tagged protein was eluted in HisPur^™^ elution buffer (50 mM sodium phosphate buffer pH 7.5, 300 mM NaCl, 300 mM imidazole). The concentrations of purified protein were quantified by Bradford assay using bovine serum albumin (BSA) as standard. For stock protein, 20% (v/v) glycerol was added; and the protein was stored at −80 °C for future use or used immediately for temperature gradient incubation specific activity assay. SDS-PAGE was performed with purified protein samples as described in the Supporting Information (Figure S6), which indicated that the protein samples have high apparent purity.

### Temperature Gradient Incubation Specific Activity Assay.

To determine thermostability, residual specific activity values were determined for CHMO variants according to previously described methods [9]. Newly purified and diluted CHMO variants (30–50 μg/mL) were incubated in sodium phosphate buffer (50 mM, pH 7.7) at various temperatures (25 °C, 30 °C, 40 °C, and 45 °C) for 10 min within PCR tubes using a gradient temperature controlled Thermocycler (Biorad). After incubation, sample activity was immediately measured at 25 °C using NADPH assay (0.1 mM NADPH, 5 mM cyclohexanone (1 M stock was dissolved in ethanol), 50 mM sodium phosphate buffer pH 7.7, 3–5 μg/mL protein).

### Steady State Kinetic Analyses for Cyclohexanone and NADPH.

Catalytic efficiencies (k_cat_/K_M_) for NADPH (Table 1) were determined according to previously described methods [9]. Reactions were performed at saturating cofactor or substrate concentrations (0.3 mM for NADPH; 5 mM for cyclohexanone). Concentrations of substrates and cofactors were changed (0.002–0.1 mM for cyclohexanone; 0.005–0.2 mM for NADPH) to allow the determination of the different steady-state kinetic parameters. The reaction was performed in 50 mM sodium phosphate (pH 7.7) at 25 °C. All the reactions were initiated by addition of an appropriate amount of the enzyme (purified protein from −80 °C, 20% glycerol stock) and the kinetic parameters were measured by monitoring the NADPH consumption at 340 nm in 96-well plate.

### Cyclohexanone Conversion Assay.

Purified and diluted CHMO variants (6 μg/mL, purified protein from −80 °C, 20% glycerol stock) were incubated in sodium phosphate buffer (50 mM, pH 7.7) with 5 mM NADPH and 5 mM cyclohexanone (1 M stock dissolved in ethanol) at incubation temperature (37 °C) in sealed glass vials. At 2, 4, and 12 h, reaction mixture was sampled. Caprolactone and cyclohexanone were extracted with an equal volume of ethyl acetate. To confirm productive NADPH oxidation, cyclohexanone conversion to caprolactone was determined for CHMO variants via gas chromatography–flame ionization detection (GC–FID) with octanol as an internal standard. Samples were analyzed using previously described GC method “KIP 5 Short” [26].

### *Ac* CHMO Homology Modeling.

The model of WT *Ac* CHMO with cofactors FAD and NADPH bound was generated with Rosetta Comparative Modeling (Rosetta CM) [37,38]. Threading templates with high sequence identity and co-crystallized cofactors were identified through a Protein BLAST (BLASTP) search of the Protein Data Bank with *Ac* CHMO as the query [39,40]. Crystal structures of CHMO from *Rhodococcus* sp. (PDB: 4RG3, 3GWD, 3GWF, 3UCL), which share 57.8% sequence identity to Ac CHMO, were selected as input models [24,41,42].The Rosetta CM protocol consisted of repeated rounds where: the target *Ac* CHMO sequence was threaded onto the template structure based on Multiple Alignment using Fast Fourier Transform (MAFFT) sequence alignment, segments of the protein structure were constructed through insertion of fragments drawn from the library provided by the templates through Monte Carlo evaluation, followed by minimization to relax the final output [43]. A total of 1500 homology modeling trajectories were completed, the output structure with the most favorable total Rosetta energy was selected as the representative model for all further analysis. Point mutations for the *Ac* CHMO variants were produced through 1000 further Rosetta docking simulations on the homology model with backbone flexibility, side chain repacking, and ligand minimizations; final models were selected by lowest total Rosetta energies.

### Molecular Dynamics Simulations.

MD simulations of the *Ac* CHMO variants were completed with PMEMD from the AMBER 18 package utilizing the ff14sb force field and 8 Å Particle Mesh Ewald real space cutoff [44–47]. Cofactor parameters were obtained from the AMBER parameter database and protonation states of titratable residues were determined with the H++ webserver [48–50]. The TLEAP program was utilized to solvate the complexes with TIP3P water molecules in a truncated octahedron with 10 Å buffer and neutralizing Na^+^/Cl^−^ counter-ions. The *Ac* CHMO systems were minimized in two stages, first with 2500 steps of steepest decent and 2500 steps of conjugate gradient where all non-hydrogen solute atoms were restrained with a 20 kcal mol^−1^ Å^−2^ force to relieve solvent clash. The second stage minimization to remove solute steric clashes was run with the same cycle settings and restraints removed. Heating from 0 K to 300 K was performed over 0.5 ns with 10 kcal mol^−1^ Å^−2^ restraints on all non-hydrogen solute atoms under isothermal-isobaric constant number, pressure, and temperature (NPT) conditions at 1 atm pressure with Langevin thermostat and 1 fs timestep. Solvent density equilibration was carried out over 5 ns with 5 kcal mol^−1^ Å^−2^ restraints on all solute atoms and an unrestrained 10 ns equilibration using 2 fs timestep to clear remaining structural artifacts followed the heating stage. Production MD trajectories were each carried out for 400 ns with 2 fs timesteps, SHAKE restraints on hydrogens, constant number, volume, and temperature (NVT) ensemble, Langevin thermostat with collision frequency 1.0 ps^−1^, and periodic boundary conditions.

### Thermostability Analysis.

Metastable conformations sampled by the *Ac* CHMO systems and representative frames for the discovered states were identified through PCA dimensionality reduction and K-Means clustering [51,52]. To understand the mechanisms behind the enhanced thermostability of GV and WT *Ac* CHMO, we compared MD trajectories of GV and WT *Ac* CHMO featurized on minimum heavy atom distance between the residues at 245 or 288 to neighboring residues having any atom within 5 Å contact. The residue positions around 245 were selected as: 62, 239, 242, 243, 244, 245, 246, 247, 250, 433, 434, 437, and 510. The residue positions around 288 were defined as: 280, 281, 286, 287, 288, 289, 290, 294, 482, 483, and 484. The distance array was standardized to zero mean and unit variance and transformed to lower dimensional space with components maintaining maximal variance through PCA. K-means clustering was performed to discretize the sampled conformations projected onto the free energy landscape of the first two PCA components into metastable states. The optimal number of clusters was selected by the elbow heuristic where clustering over a range of K values, from one to nine here, is completed and the sum of squared distances from the sample points to their assigned cluster center is computed. The value of K where the sum of squared distances decrease becomes linear is selected as optimal and indicates that increasing K further will result in over-fitting. Thermostability was evaluated through the metric of Rosetta residue energies, a score quantifying the favorability of van der Waals, electrostatic, and geometric interactions that functions as a surrogate for predicted stability, summed over all residues within 5 Å of the mutated positions [53]. 200 frames were extracted from the most populated metastable state for each sample and scored with Rosetta, the conformation with minimum distance to the cluster center was chosen for illustration.

MDTraj and cpptraj were utilized for trajectory analysis, data processing was completed with the NumPy and scikit-learn packages [54–57]. PyMol was used to illustrate the structures [58].

#### Bioinformatic Analysis.

Homologous sequences to WT *Ac* CHMO were identified through BLASTP search over the non-redundant protein sequence database [39]. Sequences were filtered on the criteria of query coverage over 70% and sequence identity between 40% and 97%, resulting in 998 hits. Multiple sequence alignment (MSA) of the matched sequences to the template *Ac* CHMO was completed with MAFFT [43]. Residue frequencies for each of the 20 canonical amino acids were computed at every position in the multiple sequence alignment.

## 5. Conclusions

In summary, we established a high-throughput screening method to select for thermostable variants of NADPH-dependent oxidoreductases. This growth-based approach utilizes a recently established *E. coli* selection strain MX203 and applies additional selection criteria for the identification of enzyme variants with enhanced thermostability. Through the selection of a random library of CHMO variants at a 42 °C incubation temperature, we identified a pair of subtle mutations, A245G and A288V, with a pronounced impact on protein stability. These mutations greatly improved residual specific activity and substrate conversion at elevated reactions temperatures. Additionally, the pair of mutations exhibited a synergistic stabilizing effect when combined with a previously identified single mutant T415C. Currently, the platform is limited to the temperature ranges tolerated by the *E. coli* selection strain. Future work may extend this approach for selection at higher temperatures to identify biocatalysts with even greater thermostability.

## Supplementary Material

Supplemental Info

## Figures and Tables

**Table 1.** Apparent kinetic parameters of CHMO WT and GV *.

NADPH					Cyclohexanone	

Enzymes	K_M_ (μM)	k_cat_ (s^−1^)	k_cat_/K_M_ (μM^−1^s^−1^)	K_M_ (μM)	k_cat_ (s^−1^)	k_cat_/K_M_ (μM^−1^s^−1^)

Wild type	36.6 ± 8.36	13.5 ± 0.55	0.37	4.09 ± 1.81	12.9 ± 0.86	3.15
GV	43.3 ± 20.9	9.67 ± 1.23	0.22	5.64 ± 0.48	11.6 ± 1.55	2.05

* The steady-state kinetics of CHMO reacting with various concentrations of cyclohexanone (0.002–0.1 mM) and NADPH (0.005–0.2 mM) were performed at pH 7.7 and 25 °C. The second substrate NADPH or cyclohexanone was added at saturation (0.3 mM for NADPH and 5 mM for cyclohexanone). Kinetic parameters are the average of three or two independent experiments where values after ± represent one standard deviation for NADPH or cyclohexanone, respectively.
