# Supplementary material for: In Vivo, High-Throughput Selection of Thermostable Cyclohexanone Monooxygenase (CHMO)
Source: Catalysts. Author manuscript; Available in PMC 2023 Aug 25. (PMC10453637; doi:10.3390/catal10080935)
Supplement: Supplemental Info [file NIHMS1919505-supplement-Supplemental_Info.pdf]

catalysts

Supplementary Materials

In Vivo, High-Throughput Selection of Thermostable Cyclohexanone Monooxygenase (CHMO)

Sarah Maxel [1], Linyue Zhang [1], Edward King [2], Ana Paula Acosta[1], Ray Luo [1,2,3], Han Li [1,*]

[1] Department of Chemical and Biomolecular Engineering, University of California, Irvine;
[2] Department of Molecular Biology and Biochemistry, University of California, Irvine;
[3] Department of Materials Science and Engineering, University of California, Irvine.
* Correspondence: han.li@uci.edu;

Received: date; Accepted: date; Published: date

Supplementary Materials

Table S1: Strains and Plasmids used in this study.
Table S2: Bioinformatic Analysis of CHMO Homologs
Figure S1: Free energy landscapes for CHMO GV and WT.
Figure S2: A288 Residue Conservation
Figure S3: Conditional Residue Distributions
Figure S4: Plasmid map of pRSF *P*BAD:: *Ac chnB*, Spec^r (pLS301)
Figure S5: Agarose gel electrophoresis fragment patterns of pRSF backbone and *Ac* CHMO library insert.
Figure S6: SDS-PAGE Protein Gel

Table S1. Strains and Plasmids used in this study

Strains	Description	Reference
XL-1 blue	Cloning strain	Stratagene
BW25113	*E. coli* F-, DE(araD-araB)567, lacZ4787(del)::rrnB-3, LAM-, rph-1, DE(rhaD-rhaB)568, hsdR514	Datsenko *et al.* [1]
DH10β	Electrotransformation strain	Invitrogen
MX203	BW25113 Δ*pgi* Δ*edd* Δ*qor* Δ*udhA::kan*	Maxel *et al.* [2]
**Plasmids**	**Description**	**Reference**
pLS101	pRSF P_{BAD}::*Lb nox*, $Spec^r$	Maxel *et al.* [2]
pLS102	pRSF P_{BAD}::*TP nox* (*Lb nox* G159A-D177A-A178R-M179S-P184R), $Spec^r$	Maxel *et al.* [2]
pLS301	pRSF P_{BAD}:: *Ac chnB*, $Spec^r$	This study
pLS302	pRSF P_{BAD}:: *Ac chnB* T415C, $Spec^r$	This study
pLS303	pRSF P_{BAD}:: *Ac chnB* Error Prone-PCR library, $Spec^r$	This study
pLS304	pRSF P_{BAD}:: *Ac chnB* A245G-A288V, $Spec^r$	This study
PLS305	pRSF P_{BAD}:: *Ac chnB* A245G-A288V-T415C, $Spec^r$	This study

Abbreviations indicate source of genes: *Lb, Lactobacillus brevis, Ac Acinetobacter sp.* NCIMB 9871

Table S2. Bioinformatic Analysis of CHMO Homologs

Accession	Species	Enzyme	Seq Id
OQU95234.1	Cladophialophora immunda	hypothetical protein CLAIMM_01470	47.42
PSN68528.1	Corynespora cassiicola Philippines	FAD/NAD(P)-binding domain-containing protein	46.49
RVX72008.1	Exophiala mesophila	hypothetical protein B0A52_04606	45.39
OJJ01939.1	Aspergillus versicolor CBS 583.65	hypothetical protein ASPVEDRAFT_52754	45.76
OJJ52384.1	Aspergillus sydowii CBS 593.65	hypothetical protein ASPSYDRAFT_164733	44.46
WP_083156499.1	Mycolicibacterium moriokaense	NAD(P)/FAD-dependent oxidoreductase	43.36
BBX04960.1	Mycolicibacterium moriokaense	cyclohexanone monooxygenase	43.36
OAA37261.1	Beauveria brongniartii RCEF 3172	cyclohexanone monooxygenase	42.25
XP_025570635.1	Aspergillus ibericus CBS 121593	cyclohexanone monooxygenase	41.88
XP_022469048.1	Colletotrichum orchidophilum	hypothetical protein CORC01_12829	46.31
WP_019875767.1	Sporichthya polymorpha	NAD(P)/FAD-dependent oxidoreductase	43.73
XP_009158444.1	Exophiala dermatitidis NIH/UT8656	cyclohexanone monooxygenase	43.36
OTA94950.1	Hypoxylon sp. CO27-5	hypothetical protein M434DRAFT_394167	41.51
TQB70714.1	Monascus purpureus	hypothetical protein MPDQ_008125	42.8
KXH34573.1	Colletotrichum simmondsii	hypothetical protein CSIM01_11797	45.02
EXF78962.1	Colletotrichum fioriniae PJ7	hypothetical protein CFIO01_10029	45.76
KXH42764.1	Colletotrichum nymphaeae SA-01	hypothetical protein CNYM01_03787	45.94
OTA57423.1	Hypoxylon sp. EC38	FAD/NAD(P)-binding domain-containing protein	41.33
KAF2164474.1	Zasmidium cellare ATCC 36951	hypothetical protein M409DRAFT_25352	42.07
WP_159765079.1	Halovenus sp. WSH3	NAD(P)-binding domain-containing protein	42.8
KXH45247.1	Colletotrichum salicis	hypothetical protein CSAL01_06360	45.57
WP_136591278.1	Salinigranum halophilum	NAD(P)/FAD-dependent oxidoreductase	43.73
WP_136601518.1	Salinigranum halophilum	NAD(P)/FAD-dependent oxidoreductase	43.73
TAL03103.1	Porticoccaceae bacterium	NAD(P)/FAD-dependent oxidoreductase	43.54
OJJ52110.1	Aspergillus sydowii CBS 593.65	hypothetical protein ASPSYDRAFT_95945	41.14
OJJ05256.1	Aspergillus versicolor CBS 583.65	hypothetical protein ASPVEDRAFT_137723	41.7
PSQ07725.1	Halobacteriales archaeon QS_5_70_15	cyclohexanone monooxygenase	43.17
KID82798.1	Metarhizium guizhouense ARSEF 977	cyclohexanone monooxygenase	41.33
XP_014540407.1	Metarhizium brunneum ARSEF 3297	cyclohexanone monooxygenase, partial	40.59

BLASTP search with CHMO WT query identified 29 homologous sequences with native G245 and V288. The complete multiple sequence alignment of the 998 hits with gap columns removed can be downloaded at https://github.com/hanli-lab/thermostable_chmo and viewed with https://www.ebi.ac.uk/Tools/msa/mview/.

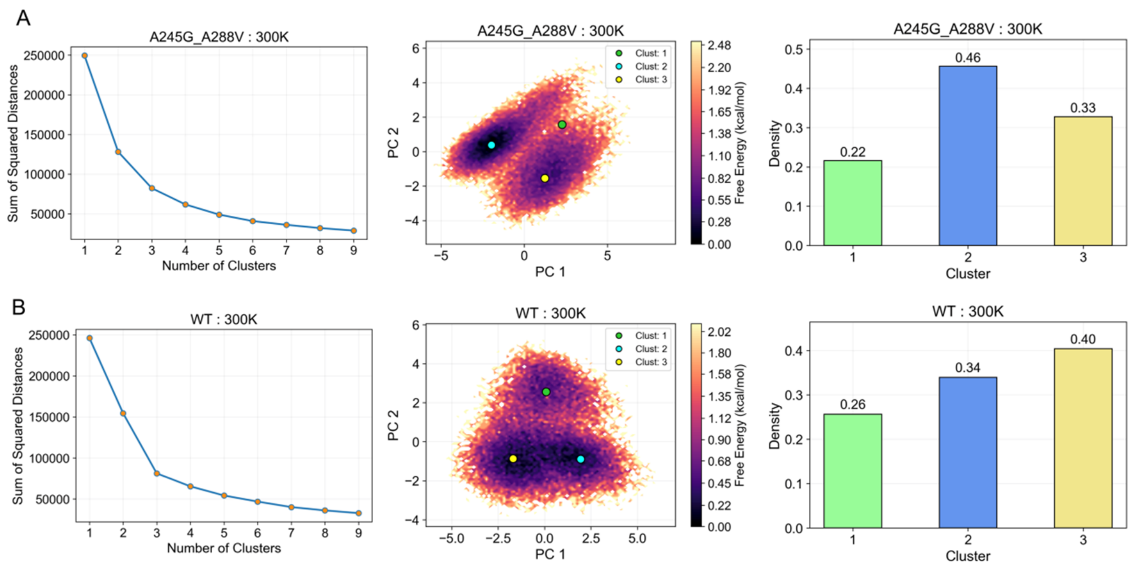

Figure S1. Free energy landscapes for GV and WT CHMO. **A)** K-means clustering elbow heuristic to determine the optimal number of clusters, free energy landscape projected on first 2 principal components and cluster centers, and cluster populations. **B)** WT CHMO K-means clustering, free energy landscape, and cluster populations.

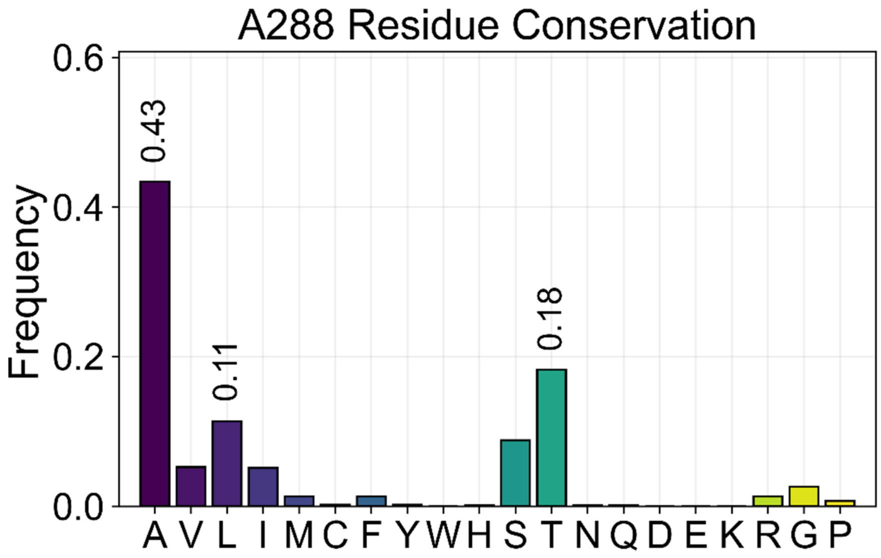

Figure S2. A288 Residue Conservation. Frequency of residues observed at position 288 from multiple sequence alignment of homologs identified through BLAST search. The native residue Ala is the most commonly found residue at 43%, while the discovered mutation to Val is identified in ~5% of homologs, indicating that this mutation has been rarely sampled in natural evolution. The distribution of residues suggests a preference for smaller amino acids at position 288.

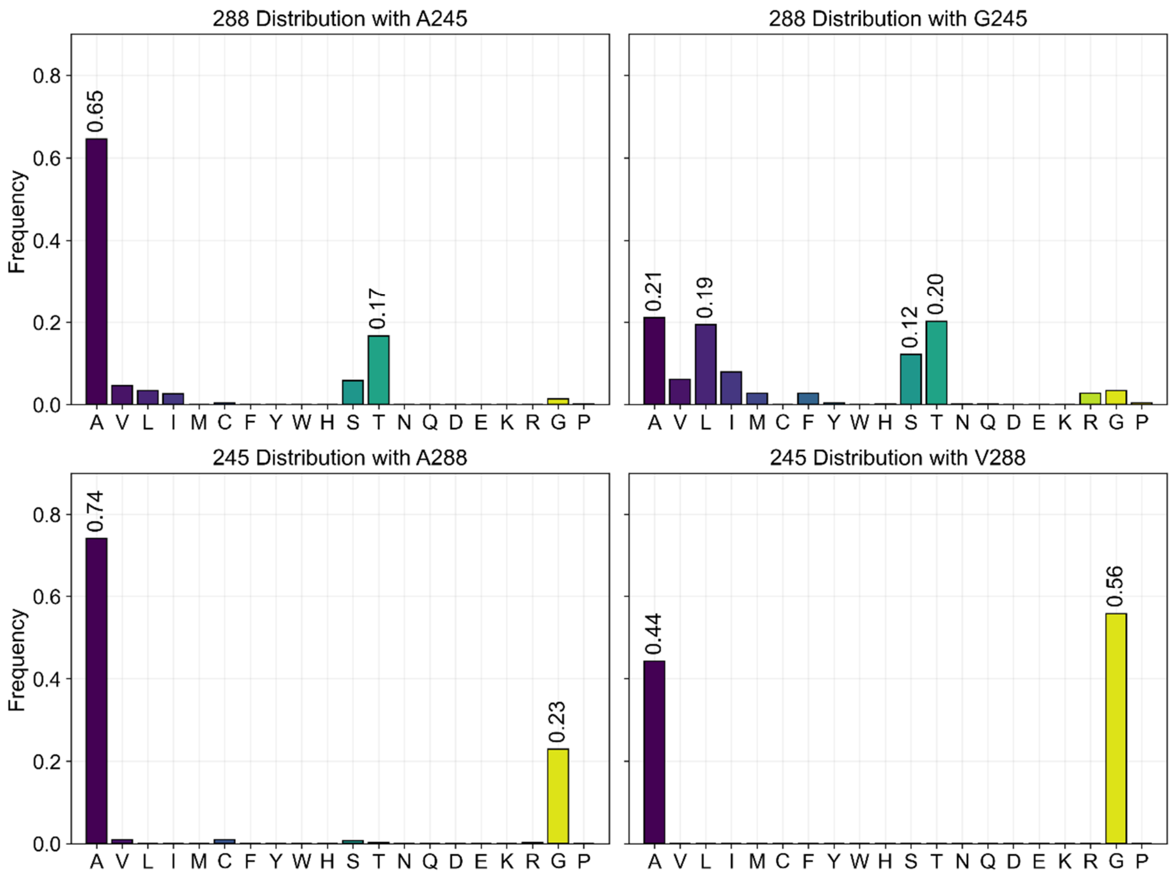

Figure S3. Conditional Residue Distributions. To evaluate the correlation of residues at position 245 and 288, we compare the conditional residue distributions at position 288 based on the presence of A245 or G245, and distributions at position 245 based on A288 or V288. Homologs with G245 show much lower frequency for A288, with the observed population dropping from 65% to 21%, and an increase in Leu and other small non-polar residues. Homologs with V288 have a more uniform distribution of Ala and Gly, while samples with A288 favor having A245. Although the conditional distributions are noticeably different, we cannot conclude that they are driven by direct co-evolution between residues at 245 and 288, this may be due simply to genetic drift.

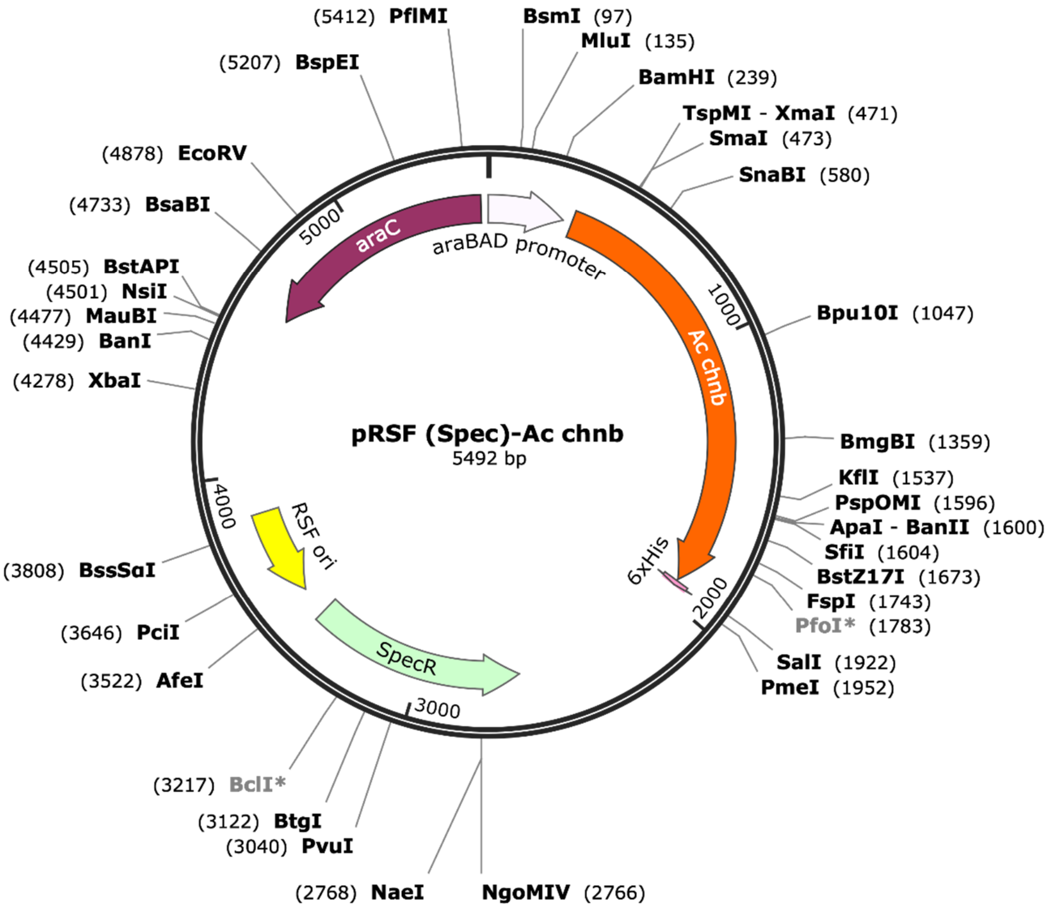

Figure S4. pLS301 plasmid map: *chnb* gene from *Acinetobacter sp*. under P_{BAD} promoter with araC repressor; spectinomycin resistant.

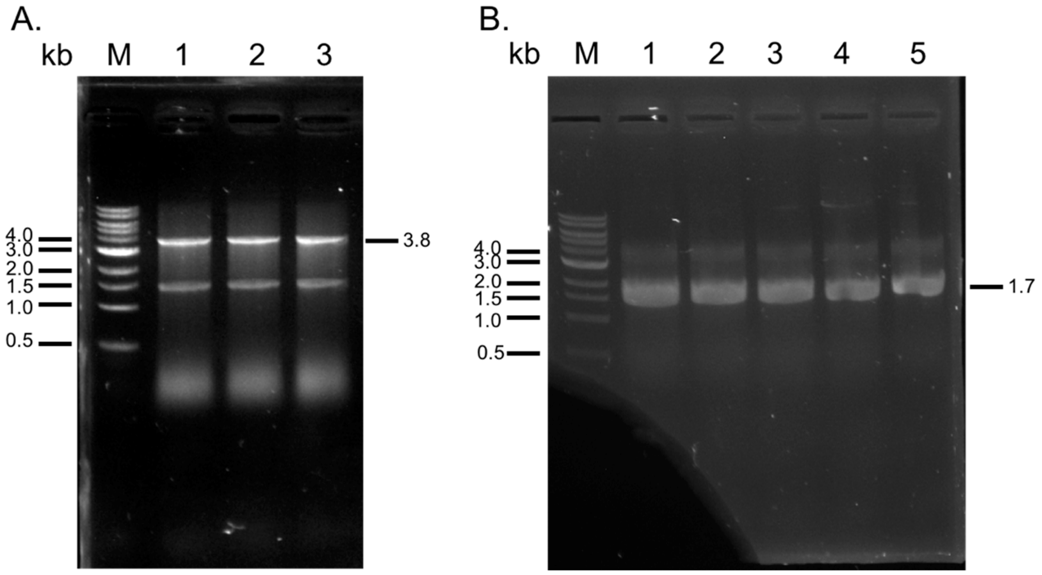

Figure S5. Agarose gel electrophoresis fragment patterns of pRSF backbone and *Ac* CHMO library insert. **A)** Lane M: 1kb ladder (NEB) as a DNA size standard; lane 1-3: pRSF backbone (3.8 kb) obtained from plasmid pLS101 by digestion with restriction enzymes BamHI-HF and SalI. **B)** Lane M: 1kb ladder; lane 1-5: target library insert (1.7 kb) that amplified via error prone PCR and digested with restriction enzymes BamHI-HF and SalI.

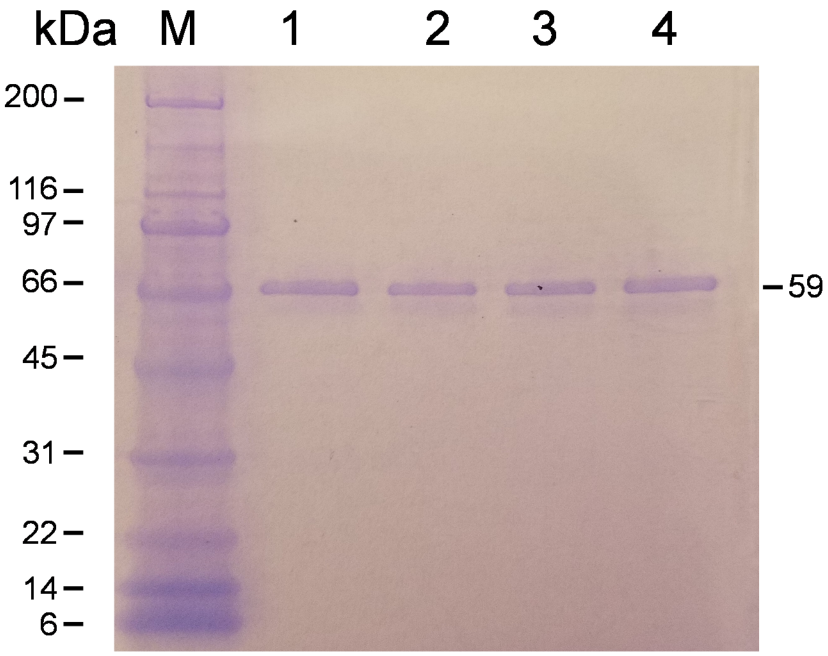

Figure S6. SDS-PAGE Protein Gel. Lane M: SDS-PAGE Standards, Broad Range (BIO-RAD) as a protein size standard; lane 1: CHMO WT obtained with plasmid pLS301; lane 2: CHMO GV obtained with plasmid pLS304; lane 3: CHMO T415C obtained with plasmid pLS302; lane 4: CHMO GV-T415C obtained with plasmid pLS305. All CHMO variants ~59 kDA.

SDS-PAGE Preparation. Samples were prepared as follows: Concentrated purified CHMO protein was diluted with HisPur™ Ni-NTA Elution Buffer and mixed in a 1:1 ratio with Laemmli Buffer, 1x Laemmli Sample Buffer with the 2- mercaptoethanol reagent (BIO-RAD). Samples were boiled at 95°C for 10 minutes before being loaded into Precast Gel (4-20%) (Mini-PROTEAN® TGX™) in 1X Tris/Glycine/SDS Buffer (BIO-RAD). Gel was run at 110V for ~45 minutes. Subsequently, Imperial Protein Stain (Thermo Scientific) was used to stain gel and visualize protein. SDS PAGE Standards (BIO-RAD, Broad Range) were prepared in parallel with CHMO protein.

References

[1] Datsenko, K. A.; Wanner, B. L. One-Step Inactivation of Chromosomal Genes in Escherichia Coli K-12 Using PCR Products. *Proc. Natl. Acad. Sci. U. S. A.*, **2000**, *97* (12), 6640–6645. https://doi.org/10.1073/pnas.120163297.

[2] Maxel, S.; Aspacio, D.; King, E.; Zhang, L.; Acosta, A. P.; Li, H. A Growth-Based, High-Throughput Selection Platform Enables Remodeling of 4-Hydroxybenzoate Hydroxylase Active Site. *ACS Catal.*, **2020**. https://doi.org/10.1021/acscatal.0c01892.

© 2020 by the authors. Submitted for possible open access publication under the terms and conditions of the Creative Commons Attribution (CC BY) license (http://creativecommons.org/licenses/by/4.0/).
